# Design and Evaluation Method of a High-Overload Test Device Based on AD-TRIZ

**DOI:** 10.3390/s25196177

**Published:** 2025-10-05

**Authors:** Peiyi Zhou, Lei Zhao, Weige Liang, Yang Zhao, Chi Li, Fangyin Tan

**Affiliations:** Naval University of Engineering, Wuhan 430033, China

**Keywords:** high overload, test, axiomatic design, innovative problem theory

## Abstract

High-overload testing equipment is a key platform for evaluating mechanical performance under extreme conditions, requiring specialized functional design to meet stringent operational demands. The current design process faces numerous challenges, including overreliance on designers’ experience, incomplete requirement analysis, and insufficient automation, resulting in suboptimal solutions. To address these issues, in this study, we propose an integrated method based on axiomatic design (AD) and the Theory of Inventive Problem Solving (TRIZ). This method systematically decomposes technical specifications, maps requirements to a structural framework, and resolves design conflicts using inventive principles. The method employs a comprehensive evaluation framework combining the analytic hierarchy process (AHP) and quality function deployment (QFD) to quantitatively assess candidate designs. It facilitates the development of efficient, standardized high-load testing equipment solutions, enhancing design reliability and innovation capabilities.

## 1. Introduction

Performance testing under high-overload conditions is a critical step in evaluating the reliability, safety, and operational capabilities of mechanical equipment and components under extreme conditions. As the foundational platform for simulating such extreme conditions, the design quality of high-overload testing equipment directly determines the accuracy and validity of test results. However, the traditional design process for high-overload testing devices heavily relies on the designer’s experience and intuition, leading to challenges such as insufficient systematic demand analysis, the severe coupling of design parameters, difficulties in generating innovative solutions, and the lack of objective quantitative evaluation standards. This often results in suboptimal design solutions, prolonged development cycles, and an inability to meet increasingly complex testing requirements.

The core design challenge of high-overload testing technology lies in accurately capturing complex dynamic parameters such as acceleration, vibration spectrum, and transient impact characteristics, while ensuring long-term structural stability and signal acquisition accuracy under extreme mechanical conditions. In recent years, scholars at home and abroad have conducted in-depth research from multiple technical perspectives. In terms of impact loading and test platform construction, Guo et al. constructed a continuous double-pulse high-overload impact test impact platform based on the sleeve-type bullet, and analyzed the protective performance of the multi-layer structure under multi-pulse based on the acceleration decay ratio [1]. Meanwhile, Tang et al. systematically addressed the effect factors of V-beam numbers, air gap, type of contact surface, external load force, periodic voltage, and gas damping on the output performance of the multi-electrothermal co-actuation device via simulation and experimental methods [2]. In terms of sensor testing methods and environmental adaptability, Chen at al. proposed a method for performing equivalent impact tests in the Hopkinson Bar overload test environment based on the Sum of Effective Impact Energy for micro-electromechanical system acceleration sensors, in accordance with the impact dynamics theory and the law of energy variation [3]. Wu et al. investigated the motion patterns of the nested test cabin in a gunpowder gas overload test device. Multiple factors during the overload impact process were explored. Under the conditions of keeping the gunpowder combustion model, the friction coefficient between the inner and outer cabins, and the mass of the cabins unchanged, the special acceleration curve and its frequency spectrum and the impact response spectrum of the pseudo-velocity were analyzed [4]. In terms of materials and structure, Sun et al. designed and prepared a novel cast explosive. Using a split Hopkinson pressure bar combined with Brazilian disk testing and digital image correlation, they characterized the dynamic properties of the explosive at moderate strain rates. From the perspective of deformation characteristics, the research results provide important references for the design and engineering applications of high-overload-resistant cast explosives. It is worth noting that extreme environmental conditions such as overload, vibration, corrosion, high pressure, high temperature, and radiation have a significant impact on sensor performance [5]. Zhang et al. considered that extreme environmental conditions such as overload, vibration, corrosion, high pressure, high temperature, and radiation may affect sensor performance up to the point of failure. They argued that compared to the overload and vibration resistance achieved through structural design, the application of sensors under extreme high-temperature and radiation conditions presents greater challenges [6].

However, although existing research has made progress in many areas, it still lacks systematic design theory support and method integration, making it difficult to fundamentally solve core problems in the design of high-overload testing devices, such as strong coupling, insufficient innovation, and a lack of evaluation systems. First, the design process relies excessively on the experience and knowledge of designers, lacking standardized procedures and systematic control, resulting in design outcomes that are strongly correlated with the experience level of personnel [7]. Second, the functional requirement analysis process is incomplete. The traditional “decomposition–design–reconstruction” process often uses a single or a few technical indicators as benchmarks, mechanically decomposing structures while ignoring interdependent factors, and treating functional implementation as a linear superposition, resulting in poor balance in design solutions [8,9,10]. Finally, the design process lacks automation and portability. When technical specifications undergo significant changes due to updates, the design process must be modified to the same extent and cannot be automatically updated or iterated [11]. Standardization and informatization levels lag behind those of commercial product design. These systemic bottlenecks, resulting from reliance on experience, one-sided functional analysis, and lack of automation, severely limit the reliability of high-overload test equipment in critical scenarios such as re-entry into the atmosphere and hypersonic penetration for new spacecraft. To systematically address these issues, standardized, proceduralized, and normalized design methods are currently widely adopted. The existing design methods primarily fall into the following categories:

1. Axiomatic Design (AD): AD was proposed by Professor Nam P. Suh of MIT, with its core principle being the use of two fundamental axioms to guide the engineering design process [12]. The Independence Axiom emphasizes that functional requirements must be mutually independent, ensuring that changes in design parameters only affect a single functional requirement, thereby avoiding the uncontrollability caused by system coupling. The Information Axiom requires the design process to minimize information uncertainty, using probabilistic models to assess the reliability of design solutions and achieve efficient decision-making. Axiomatic design employs a mapping method between functional and physical domains, using a “zigzag” decomposition path to convert user requirements into executable technical parameters. This approach is suitable for structured complex systems and enhances design traceability [13].
2. Theory of Inventive Problem Solving (TRIZ): TRIZ originated from the systematic study of global patents by Soviet inventor Genrich Altshuller, aiming at distilling universal laws of technological innovation [14]. This theory focuses on technical conflicts, abstracts engineering problems into contradictions between improving parameters and deteriorating parameters, and provides solutions through a contradiction matrix matching forty inventive principles. The concept of an idealized final result drives designers to utilize system resources to maximize functionality and minimize material usage, such as by separating principles to resolve physical contradictions. The Theory of Inventive Problem Solving also introduces a material–field model to analyze component interactions, supporting interdisciplinary problem solving, particularly in breaking through traditional thinking constraints and accelerating concept generation [15].
3. Robust Design: Robust design is an engineering method that aims to improve consistency and reliability by optimizing product parameters and system architecture to make functional performance insensitive to uncontrollable noise factors. It originates from Taguchi’s philosophy of quality, which emphasizes embedding robustness at the conceptual stage [16].
4. Design For Six Sigma (DFSS): DFSS is a systematic framework driven by customer needs, which achieves Six Sigma quality levels through the DMADV process. Various types of systematic design methods have different scopes of application [17].

Given the unique requirements of high-overload testing equipment, existing mainstream methods exhibit systemic limitations, as shown in Table 1. While robust design can enhance noise immunity through parameter combination optimization, it struggles to overcome structural innovation bottlenecks, particularly in terms of new materials or adaptability to extreme environments [18]. Meanwhile, Six Sigma design ensures rigor through phased processes such as IDOV or DMADV, but its strict control mechanisms significantly slow down response times in scenarios of rapid technological iteration or uncertainty, leading to reduced design efficiency [19]. Furthermore, while the quantitative framework of axiomatic design offers structural decoupling advantages, it fails in novel impact environments due to the lack of historical data benchmarks. A typical example is the strong coupling issue between equipment dimensions and interface layout, which cannot be fully resolved through independence axioms and instead increases design redundancy. However, while the TRIZ method can resolve local technical conflicts through a contradiction matrix, its lack of system requirement decomposition capabilities results in fragmented innovation solutions. For example, engineering parameter conflicts require manual intervention to match solutions, making it difficult to achieve overall design coordination [20]. Therefore, while each of these methods has its own advantages, they all expose core deficiencies in complementary capabilities under high-dynamic, high-complexity overload testing scenarios.

This study introduces a fusion method based on AD and TRIZ. By systematically decomposing and mapping user requirements using AD theory, a clear design matrix is established to guide the design process and identify coupling conflicts. Subsequently, conflict resolution tools from TRIZ theory are applied to provide innovative principles and solutions for eliminating design contradictions. Additionally, this study combines quality function deployment (QFD) and the analytic hierarchy process (AHP) to construct a comprehensive quantitative evaluation system, enabling objective assessment and selection of optimal solutions among multiple candidate options. This study aims to elucidate this integrated design method and process, and through practical application, demonstrates that this method can significantly enhance the design efficiency, innovation, and decoupling degree of high-overload test equipment, providing a scientific and reliable theoretical basis and practical framework for the R&D of high-end testing equipment.

## 2. Evaluation System of High-Overload Test Device

The structural design and performance evaluation of high-overload environment testing devices aim at establishing standardized design procedures for such equipment in extreme conditions. This involves optimizing component testing capabilities, unit layout configurations, and physical constraints to enhance measurement accuracy and ensure device and data security, while extending the overall lifespan of the testing apparatus [30,31,32]. These devices operate within a complex physical field environment characterized by low-frequency motion, medium-frequency vibrations, and high-frequency impacts. The structural design process primarily focuses on the following key aspects [33,34].

1. Physical characteristics of test units: Because the test device is usually arranged in the object moving at high speed, its arrangement position is specially designed or replaced by other devices. The structure after the installation of the test device needs to be consistent with the specified functions of the various actions without the test device.
2. Test characteristics of test units: The test unit must complete its tasks under high-overload conditions, analyzing various indicators of high-speed motion characteristics from the equipment under test to form a description of the high-load motion process [35]. As this testing device requires low-, medium-, and high-frequency measurement and recording of motion data, it necessitates the use of sensors with different performance characteristics. The selection and combination of sensor types should be determined through analysis based on the specific testing functions required.
3. Power supply characteristics of test units: During the operation of the tested equipment, the test unit independently measures and records test data while functioning as a self-starting unit through its switching mechanism. Simultaneously, all sensors and storage units must iteratively record and store data while automatically verifying the validity of their information. This requires high-density, large-capacity power supplies to ensure continuous power supply and guarantee the completion of specified functions within a defined timeframe [36]. However, since the power supply unit is an integral part of the test unit, it necessitates balanced design considerations based on size and functional requirements to achieve optimal configuration.
4. Test data transfer and storage characteristics of units: Given the same application requirements as power supply characteristics, during various testing processes, test data must be stored in internal storage units and transmitted to external analysis software at appropriate times [37]. Therefore, it is necessary to plan suitable storage unit specifications based on basic testing requirements, while designing universal interfaces and transmission/charging protocols to meet daily data and charging needs during routine operations.

Corresponding to the overall design requirements, it is necessary to focus on the above four aspects of centralized design, combined with standardized design methods for analysis. The design process needs to combine kinematics, mechanism dynamics, microelectronics technology, and data processing technology.

## 3. Overload Test Device Design Process

Based on the four major research dimensions identified in the analysis, we implement a multi-process and multi-level design methodology that integrates benchmark requirements. The core design philosophy employs “coupling–decomposition” and “spiral progression” principles to systematically refine technical challenges. By translating theoretical concepts into engineering solutions, we address non-decomposable technical complexities through practical implementation, ultimately developing actionable design solutions [38].

### 3.1. Functional Requirement Analysis

In the design process, the first step is to establish specific performance requirements for the test equipment. By using the functional requirements of different types of high-load test equipment as input conditions for system design, we apply functional decomposition theory and axiomatic design theory to achieve the independence and decoupling of functional design [7]. This independence should be realized in both spatial and temporal dimensions. Spatial independence refers to the physical separation of components through spatial layout during implementation. Functional requirements (FRs) must correspond one-to-one with design parameters (DPs) to avoid coupling (i.e., modifying one DP does not affect other FRs). If spatial independence cannot be achieved during the design phase, temporal independence must be ensured, with process variables (PVs) controlled independently in a time series to prevent interference between different stages of operation. When both types of independence cannot fully meet all functional requirements, additional components must be introduced to achieve specialized functional design. In the field applied in this paper, spatial independence means that various testing requirements must be met independently; for example, overload testing capability (overload sensor), attitude testing capability (attitude sensor), and corresponding functions must be performed by independent execution units. However, due to the integration of microelectronics technology, these components are integrated into a common module. Therefore, designers must plan the execution sequence to ensure the parallel execution and simultaneous recording of the test object’s state, which is a logical temporal execution sequence. This must be planned during the design process.

### 3.2. Demand–Design Mapping Analysis

This paper establishes a demand–design mapping quality model to establish an application demand quality framework. First, we conduct an importance analysis of functional requirements using weighting evaluation methods and then rank these requirements based on their significance to form a prioritized list. For each refined requirement, we propose implementable technical solutions with quantifiable [8] implementation capabilities. Different functional implementations may exhibit either single-function mapping or coupling mapping relationships. In the case of coupling mapping, we analyze the criticality of different design implementations in fulfilling functional requirements and determine whether there are interdependencies among these designs. Simultaneously, this process enables the comprehensive evaluation of multiple design solutions derived from requirement–design interactions, providing theoretical foundations for the further optimization and decoupling of designs.

### 3.3. Theory of Inventive Problem Solving and Functional Analysis

Traditional methods typically rely on past design experience to solve emerging technical issues and address technical conflicts and design coupling issues that arise in the demand-driven design process [39]. However, this method carries significant uncertainties, heavily depending on designers’ expertise and subjective judgment. It fails to comprehensively reflect problem dimensions and may hinder practical solutions. Therefore, in this study, we employ an innovative problem-solving methodology within existing technical domains to accelerate resolution and system logic standardization. The Applied Invention Problem-Solving Theory is adopted for analyzing technical conflict resolutions. This theory identifies 39 engineering parameter conflicts in functional implementation design, constructing a problem-solving matrix by integrating these parameters with 40 inventive principles. Through this matrix, we explore innovative technical solutions and decoupling designs [9].

### 3.4. Design Scheme Evaluation Method for High-Load Testing Equipment

**Figure 1:**
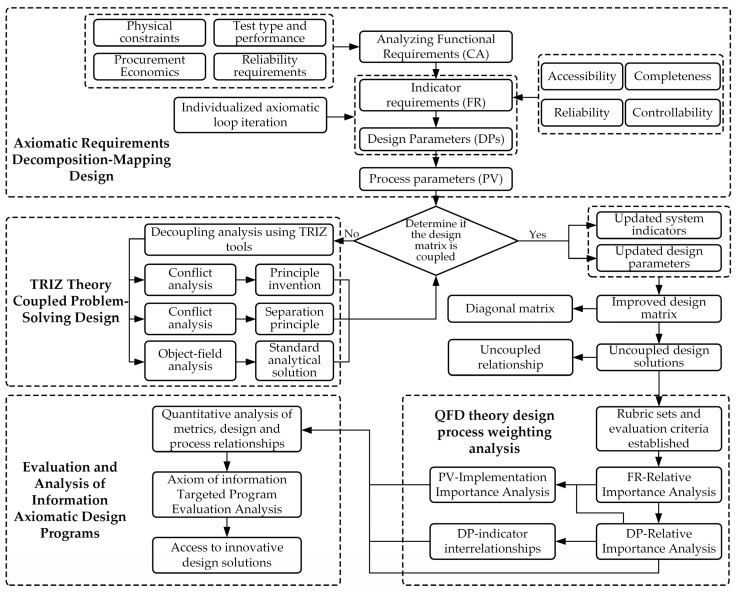
Flow chart of design and evaluation method of high-overload test device. (The dashed boxes indicate the primary methodology employed at each stage: AD for decomposition and mapping, TRIZ for conflict resolution, and AHP/QFD for evaluation).

Given the potential for multiple design schemes, establishing an objective and effective evaluation system to assess various design schemes is a critical step in the design process. In this paper, we propose a comprehensive fuzzy evaluation method for quantifying design schemes. Its fundamental analytical principle involves converting qualitative indicators that cannot be quantified into quantifiable data. Through functional analysis and the requirement-to-design mapping process, various indicators are identified, their importance is assessed, and their impact is quantified. This establishes a data-driven, clear evaluation framework to complete the evaluation process. The analysis outputs from each stage serve as inputs for subsequent stages. The quality of design analysis in each stage influences the overall integrity of the final design, and the entire process is an iterative optimization cycle. The workflow diagram is shown in Figure 1. The AD process is responsible for system decomposition and mapping, while the TRIZ process is specifically applied to resolve conflicts identified during the design coupling analysis stage.

## 4. Application Examples

### 4.1. Principles for Establishing Functional Requirement Index System of High-Overload Test Device

**Figure 2:**
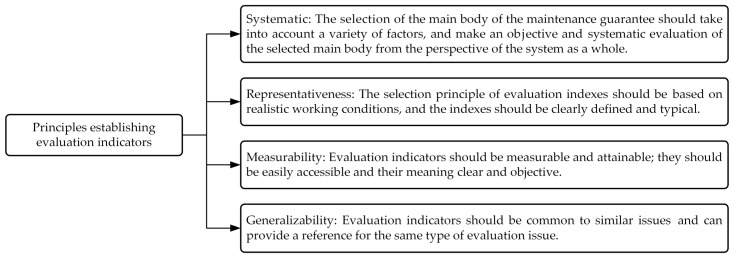
Basic principles for establishing the demand index system.

Establishing a comprehensive design indicator system that meets the functional requirements of high-overload testing equipment is a crucial prerequisite in the design process, and this system also serves as a key reference for subsequent evaluation and analysis. Currently, various typical indicators established based on requirements exhibit issues such as subjectivity, arbitrariness, and poor accuracy. While adhering to fundamental principles like scientific rigor, practicality, and operability, extensive data collection and user requirement surveys are required. After clarifying the primary tasks of supply assurance, it is essential to conduct an in-depth analysis of specific processes and key influencing factors under each task. Through thorough literature review and research report analysis, combined with qualitative and quantitative assessments, along with consulting industry experts’ professional opinions, a clear demand indicator system can be established. The basic principles are illustrated in Figure 2.

### 4.2. Functional Requirement Index System of Overload Test Device

The high-overload test device needs to record and monitor the motion state and mechanical characteristics of the equipment during each stage of operation. The specific user requirements are shown in Table 2.

Based on the axiomatic design theory, in this paper, we further decompose and map it to clarify the top-level requirements and conduct hierarchical decomposition. According to the requirements, all components of the testing device need to be installed in a fixed-shaped high-speed uncontrolled aircraft structure, which requires the realization of testing functions within limited space.

1. Physical properties (CA1)
  The system is designed to replicate the operational requirements of conventional high-speed unmanned aerial vehicles (UAVs) during launch platform operations. Its primary design features must maintain identical physical appearance to standard UAVs, with dimensional specifications clearly defined. Furthermore, its static physical characteristics should align with those of conventional UAVs. The functional requirements for these physical attributes are specifically outlined in terms of mass characteristics and center of mass positioning.
2. Test characteristics (CA2)
  Based on key performance indicators for assessing mechanism motion characteristics, critical parameters must be measured during high-speed uncontrolled flight operations: motion, attitude, vibration, and impact parameters. Motion parameters specifically capture low-speed operational patterns transmitted through launch platform mechanisms. Attitude parameters record coordinate system transformation processes during motion. Vibration and impact parameters document regular structural vibrations and transient impacts encountered during normal operation. These parameters collectively represent typical dynamics and kinematic characteristics of the launch platform, providing comprehensive operational status monitoring.
3. Battery characteristics (CA3)
  As a critical component of testing equipment, battery cells undergo frequent charge–discharge cycles due to their operational characteristics, resulting in relatively shorter lifespan than the overall system. Under normal operating conditions, battery cell longevity is significantly lower than that of the entire testing device. Therefore, to ensure the extended service life of the entire system during operation, it is essential to establish specific regulations and constraints for battery cell durability.
4. Storage cell characteristics (CA4)
  After completing various testing tasks, the test device must store test data in its internal storage unit and transmit the complete dataset to external analysis software at appropriate times. Therefore, it is essential to design suitable storage unit specifications based on fundamental testing requirements, while developing universal interfaces and transmission/charging protocols to meet daily operational data management and charging needs.

### 4.3. Requirement–Design Mapping Analysis

According to the functional requirements, the functional design is mapped to form the complete functional structure of the test device.

**Figure 3:**
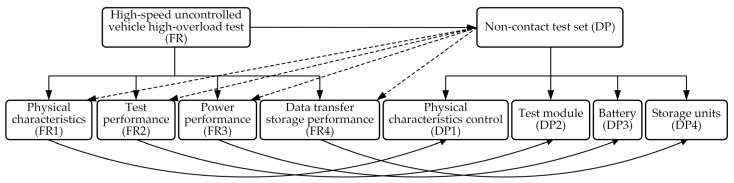
Map from layer 1 to layer 2.

**Figure 4:**
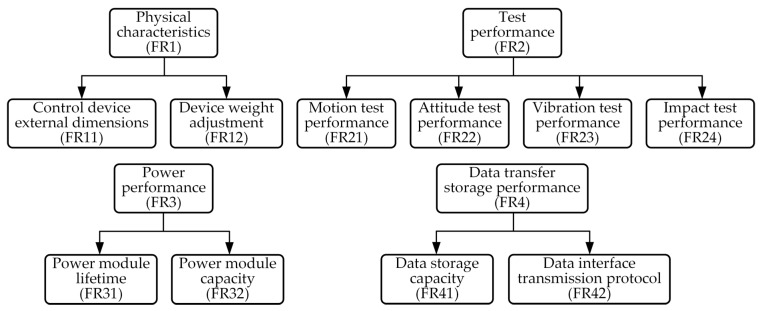
Third-level decomposition of functional domain.

**Figure 5:**
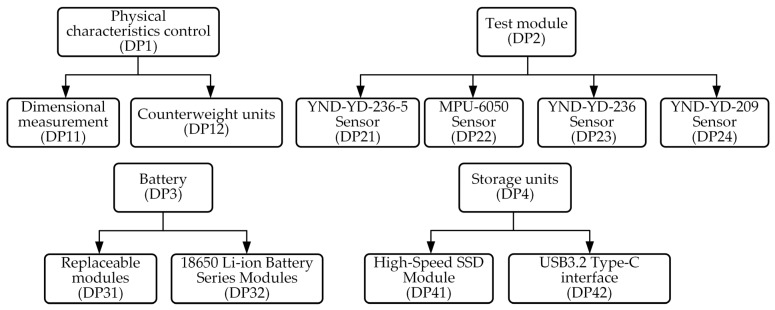
Physical domain third-level decomposition diagram.

**Figure 6:**
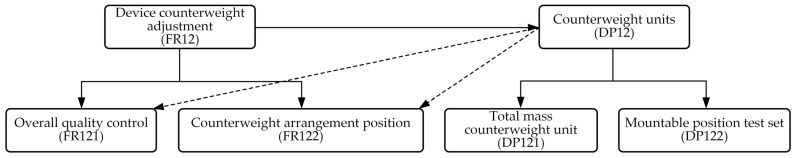
Functional domain and physical domain fourth-layer decomposition diagram.

Based on the axiomatic design principle, the requirements described in Section 3.1 can be further refined to ultimately form the test equipment functional domain (FRi) parameters shown in Figure 2. Based on these requirements, the test equipment can be developed with four core functions: physical characteristics (FR1), test performance (FR2), power supply performance (FR3), and data transmission and storage function (FR4). The corresponding physical domain includes four problem-solving modules, namely physical characteristics control unit (DP1), test unit (DP2), battery unit (DP3), and storage unit (DP4), as shown in Figure 3. Although this level of functional decomposition has partially clarified the functional domains and physical domains, further refinement is still required. Specifically, the physical characteristics control unit is primarily responsible for verifying whether the physical dimensions and mass characteristics of the test equipment meet the standards of real high-speed unmanned aerial vehicles. This requires further decomposition into equipment dimension control (FR11) and weight adjustment (FR12), with specific implementations including overall dimension control and measurement of the test equipment (DP11) and design and adjustment of the weight unit (DP12). The test apparatus primarily performs status measurements at different operational stages of the launch platform system, with the core objective of achieving optimal test performance. Based on historical data analysis, the system needs to measure four key parameters: motion (FR21), attitude (FR22), vibration (FR23), and impact parameters (FR24). To meet these requirements, four dedicated modules have been designed: the YND-YD-236-5 motion parameter sensor (DP21) for motion parameter detection, the YND-YD-236 vibration sensor (DP23) for vibration analysis, the YND-YD-209 impact sensor (DP24) for impact response assessment, and the YND-YD-6050 attitude sensor (DP22). As a critical component ensuring system reliability, the battery module must have sufficient charge/discharge cycle life (FR31) and standard energy capacity (FR32). Quantitative specifications have been established for both parameters. The current configuration uses replaceable 18,650 lithium batteries (DP32) connected in series to form battery cells (DP31), with specific models selected based on operational requirements. The storage unit must ensure that test data is fully preserved and retained without loss (FR41) and that data is promptly transmitted after testing (FR42). Therefore, a high-speed SSD storage medium (DP41) is used for data recording, and the data/charging interface is configured to USB 3.2 Type-C specifications (DP42), with the specific mapping relationships shown in Figure 4 and Figure 5. By decomposing the three functional domains and physical domains, the high-overload test device has achieved its basic physical implementation. However, to conduct an in-depth analysis of the weight adjustment design process (FR12), a fourth-level decomposition is required. As shown in Figure 6, this weight adjustment process can be decomposed into overall quality control of the test equipment (FR121) to meet the quality requirements of standard high-speed drones. Under this quality standard, a weight distribution positioning scheme (FR122) was simultaneously designed. This ultimately formed the specific functional weight unit mass (DP121) and its installation location within the test equipment (DP122), thereby establishing the overall functional design mapping system for the high-load equipment. A design coupling analysis was conducted on the developed mapping structure, with the product design matrix shown in Equation (1) [12]:

(1) $$\overset{\left[\mathrm{FR}\right]}{\overset{︷}{\left[\begin{array}{c} \mathrm{FR}11\\ \mathrm{FR}121\\ \mathrm{FR}122\\ \mathrm{FR}21\\ \mathrm{FR}22\\ \mathrm{FR}23\\ \mathrm{FR}24\\ \mathrm{FR}31\\ \mathrm{FR}32\\ \mathrm{FR}41\\ \mathrm{FR}42 \end{array}\right]}}=\overset{\left[A\right]}{\overset{︷}{\left[\begin{array}{ccccccccccc} X & 0 & 0 & 0 & 0 & 0 & 0 & 0 & 0 & 0 & X\\ 0 & X & X & 0 & 0 & 0 & 0 & 0 & 0 & 0 & 0\\ 0 & 0 & X & 0 & 0 & 0 & 0 & 0 & 0 & 0 & 0\\ 0 & 0 & 0 & X & 0 & 0 & 0 & 0 & 0 & 0 & 0\\ 0 & 0 & 0 & 0 & X & 0 & 0 & 0 & 0 & 0 & 0\\ 0 & 0 & 0 & 0 & 0 & X & 0 & 0 & 0 & 0 & 0\\ 0 & 0 & 0 & 0 & 0 & 0 & X & 0 & 0 & 0 & 0\\ 0 & 0 & 0 & 0 & 0 & 0 & 0 & X & 0 & 0 & 0\\ 0 & 0 & 0 & 0 & 0 & 0 & 0 & 0 & X & 0 & 0\\ 0 & 0 & 0 & 0 & 0 & 0 & 0 & 0 & 0 & X & 0\\ 0 & 0 & 0 & 0 & 0 & 0 & 0 & 0 & 0 & 0 & X \end{array}\right]}}\overset{\left[\mathrm{DP}\right]}{\overset{︷}{\left[\begin{array}{c} \mathrm{DP}11\\ \mathrm{DP}121\\ \mathrm{DP}122\\ \mathrm{DP}21\\ \mathrm{DP}22\\ \mathrm{DP}23\\ \mathrm{DP}24\\ \mathrm{DP}31\\ \mathrm{DP}32\\ \mathrm{DP}41\\ \mathrm{DP}42 \end{array}\right]}}$$

Here, [FR] represents the functional requirements matrix; [DP] denotes the design parameter vector; and [*A*] stands for the mechanism design matrix. The fact that matrix [*A*] is neither diagonal nor triangular indicates an integrated coupling design approach. Specifically, the test device’s dimensions must conform to specified error ranges while maintaining a streamlined shape without protrusions on its exterior. However, the data interface requires openings at appropriate positions on the outer wall to connect external devices, which compromises structural integrity, structural strength, and rigidity. These conflicting requirements create mutual interference with functional output specifications.

Similarly, the overall quality control of the testing device is also affected by the weight position and the number of mass blocks, which exhibit a certain coupling effect in the design. Therefore, it is necessary to further analyze the correlation design of the coupling matrix, combine the problem-solving theory to decouple the design, and finally form an optimized design scheme.

### 4.4. Theory of Inventive Problem Solving and Function Analysis

Based on TRIZ theory’s 39 standard engineering parameters shown in Appendix A, conflict matrix, and 40 inventive principles, the design process involves converting specific technical conflicts into standardized engineering parameters. By referencing parameter codes in the conflict matrix, designers identify corresponding inventive principles to guide innovative design. This approach allows structural decoupling through separation principles or simplifies complex designs by adding independent fields or objects via material–field models. The design ultimately achieves innovative decoupling solutions. In this project, two sets of conflicting parameters were identified:

1. During the design of the test device, it is essential to ensure that its structural form and dimensions closely resemble those of standard high-speed unmanned aerial vehicles (UAVs), while simultaneously incorporating wired data/power interfaces at appropriate positions. This creates a conflicting requirement between Technical Feature Parameter No. 9 (Speed) and Technical Feature Parameter No. 32 (Ease of manufacture) in the engineering parameter table.
2. The overall quality control and the arrangement position of the counterweight are converted into technical characteristic parameters No. 2 (Weight of stationary object) and No. 36 (Device complexity) in the engineering parameter table, forming a conflict.

The conflict matrix is searched by technical characteristic parameters, as shown in Table 3.

The inventive principles recorded in Table 4 are listed in Table 3.

The following two optimization principles were selected to resolve technical conflicts arising from overall size control and measurement (DP11) and the USB3.2 Type-C specification (DP42): Principle 5 for the unified processing of external interfaces through a single standardized interface, enabling all external functions to be integrated into one port while optimizing internal wiring layout, and Principle 35 for parameter variation optimization. The application of Principle 5 is based on the functional similarity of external interfaces rather than their physical form. This is achieved through the standardization of the USB 3.2 Type-C interface, which optimizes internal wiring layout, avoids redundant design, and aligns with the core principle of TRIZ’s “unified handling of similar components.” If there are significant functional differences between interfaces, Principle 6 may be more applicable; however, in this design, all interfaces are of the low-voltage data/power type, so Principle 5 takes precedence. The latter principle positioned interfaces at the bottom/forefront of high-speed unmanned aerial vehicles (UAVs), eliminating the option of side-wall mounting. Interface locations were strategically positioned to avoid contact with other mechanical components, symmetrically arranged relative to mounting fixtures, and designed with recessed ports to theoretically achieve structural coupling. For the technical conflicts between the total weight of the counterweight unit (DP121) and installation positions within testing equipment (DP122), Principle 1 and 35 were applied. The division principle (Principle 1) split the counterweight unit into front and rear sections, while multiple counterweight plates replaced monolithic weights to enable multi-level adjustability. This resulted in two distinct design solutions:

1. Scheme I: Various sensors are installed at the front position of the high-speed uncontrolled aircraft. The entire structure is installed from the front fairing mounting position and secured with threads at the fairing mounting position. The panel is equipped with switch/data/power interfaces. The panel is recessed into the high-speed uncontrolled aircraft. During testing, the counterweight fairing is screwed on to protect the panel. After testing, the high-speed uncontrolled aircraft is recovered, the counterweight fairing is unscrewed, and data is read from the panel via the data cable. The main structure of the test mechanism is the mechanism frame, made from aluminum alloy rods. Various sensors, data storage devices, and battery packs are installed on the frame. The impacts and vibrations experienced by the high-speed uncontrolled aircraft are transmitted from the front to the frame, and then from the frame to the sensors.
2. Scheme II: Sensors are mounted on the bottom of the aircraft. The test core with various sensors and circuit boards is installed on the bottom of the aircraft, with the battery pack positioned adjacent to the test core. The sensor array is installed on the bottom of the high-speed uncontrolled aircraft, followed by the assembly of the aircraft. The impacts and vibrations experienced by the high-speed uncontrolled aircraft are transmitted from the bottom contact points to the skeleton, and then from the skeleton to the sensors.

Both schemes can meet the current test tasks, but it is impossible to evaluate their advantages and disadvantages subjectively. It is necessary to use evaluation theory to quantify each index and form the optimal design scheme.

### 4.5. Evaluation and Analysis of Design Schemes Based on Comprehensive Evaluation Method

In this paper, we use the indicator analysis framework established in Section 4.2 to conduct a quantitative weight analysis of the evaluation system, thereby constructing a numerical analysis framework to optimize multiple design schemes developed during the design process. We utilize the analytic hierarchy process (AHP) for quantifying indicators, followed by comprehensive evaluation through QFD theory and assessment methodologies. The QFD approach establishes intuitive and complete weightings and interrelationships among various indicators through mapping requirements to functional specifications. The determination of indicator weights in the QFD process requires the integrated application of both subjective and objective analytical methods.

#### 4.5.1. Determination of Index Weight Based on Analytic Hierarchy Process

Ten experts were invited to participate in analytic hierarchy process (AHP) scoring to ensure the objectivity and authority of the evaluation. The expert panel consisted of three professors specializing in mechanical design and extreme environment engineering, four senior engineers with over ten years of experience in test equipment development, and three PhD researchers focused on design methodology. This composition ensures a balance between theoretical knowledge and practical experience.

Firstly, the analytic hierarchy process was used to invite m experts to score each category of *n* design requirements. The scoring criteria are shown in Table 5, and *m* weight judgment matrices can be obtained. The weighted coefficients were calculated by using the sum method, and the weighted preference of m experts was obtained.

First, the weight judgment matrix ${\bar{A}}_{m}$ is normalized by column, and the elements in the matrix satisfy [40]:

(2) $${\bar{a}}_{mij}=\frac{a_{mij}}{\sum_{i=1}^{n}a_{mij}}\left(i,j=1,2,\cdot \cdot \cdot ,n\right),$$

(3) $${\bar{A}}_{m}=\left[\begin{array}{cccccc} 1 & {\bar{a}}_{m12} & L & {\bar{a}}_{m1j} & L & {\bar{a}}_{m1n}\\ {\bar{a}}_{m21} & 1 & L & L & L & a_{m2n}\\ M & L & 1 & {\bar{a}}_{mij} & L & M\\ {\bar{a}}_{mj1} & L & {\bar{a}}_{mji} & 1 & L & M\\ M & L & L & L & 1 & M\\ {\bar{a}}_{mn1} & {\bar{a}}_{mn2} & L & L & L & 1 \end{array}\right],$$

where $0<{\bar{a}}_{mij}<1$, which ultimately leads to the normalized matrix ${\bar{A}}_{m}$. Averaging it by rows gives the weight coefficients:

(4) $$\omega_{mi}=\frac{\sum_{j=1}^{n}{\bar{a}}_{mij}}{n}.$$

From this, the vector of weight coefficients for the *m*th expert’s score is computed, $W_{m}$. By analogy, the vector of weight coefficients for the other experts is computed, ${\bar{A}}_{m}$. The eigenroots of the normative matrix $\lambda_{m\max}$ are calculated [40].

(5) $$S_{m}=\left[s_{m1}\;s_{m2}\;\cdot \cdot \cdot \;s_{mn}\right]={\bar{A}}_{m}\cdot W_{m},$$

(6) $$\lambda_{m\max}=\left(\frac{s_{m1}}{w_{m1}}+\frac{s_{m2}}{w_{m2}}+\ldots +\frac{s_{mn}}{w_{mn}}\right)/n,$$

where *n* is the judgment matrix order. Then, the consistency index *CI* is calculated and the consistency ratio *CR* is judged [40]:

(7) $$CI=\frac{\lambda_{m\max}-n}{n-1},$$

(8) $$CR=\frac{CI}{RI}.$$

Average random consistency index *RI*: The smaller the *CI*, the better the consistency of the evaluation results; when *CR* < 0.1, it has a more satisfactory consistency; if not, we need to correct the judgment matrix. *W_m_* is the evaluation result of a single expert for the evaluation index, and the subjectivity will have a certain impact on the final result. The fusion of the evaluation results of multiple experts can be obtained as a multi-expert weight vector *W* [40].

(9) $$W=\left(B_{1}\cdot W_{1}+B_{2}\cdot W_{2}+\cdot \cdot \cdot +B_{m}\cdot W_{m}\right)/m$$

The average random consistency index *RI* for a 10th-order matrix is 1.49. A *CR* value less than 0.1 is considered to indicate satisfactory consistency. All expert evaluations in this study underwent this consistency check, and any inconsistent judgments were returned to the experts for revision until *CR* < 0.1 was achieved.

#### 4.5.2. Comprehensive Utilization of Index Weights Using QFD Method

A triple approach combining information entropy, AHP, and QFD was employed to conduct a comprehensive evaluation of the design proposal. The evaluation results were subjected to comparative analysis to derive the overall assessment outcome for this proposal. In Equation (9), *B_m_* is the evaluation weight of different experts in the expert group. *B_m_* can be specifically selected according to the actual situation; in this design, it is considered to be the same as the weight of the experts: m = 10; n = 10. Finally, we can obtain the value of the weight of the design requirements. Then, in assessing the quality of the house to describe a single design requirement, different technical measures are used for the existence of design requirements for a very weak, weak, general, close, and very close relationship, respectively, with 1, 3, 5, 7, and 9 used to indicate the degree of this relationship (with 2, 4, 6, and 8 in between). The relative relationship indicator *r_sj_* is used to indicate its specific value. In this design process, combined with the design demand weights *W_i_*, the technical importance *A_j_* and technical weights *B_j_* are calculated [40], and the formula in the design demand and technical measures shows a one-to-one correspondence: *k* = *n* = 10; the specific calculation process is as follows:

(10) $$A_{j}=\sum r_{sj}\times W_{i}\left(s,i=1,2,\ldots ,n;j=1,2,\cdot \cdot \cdot ,k\right),$$

(11) $$B_{j}=\frac{A_{j}}{\sum A_{j}}.$$

**Figure 7:**
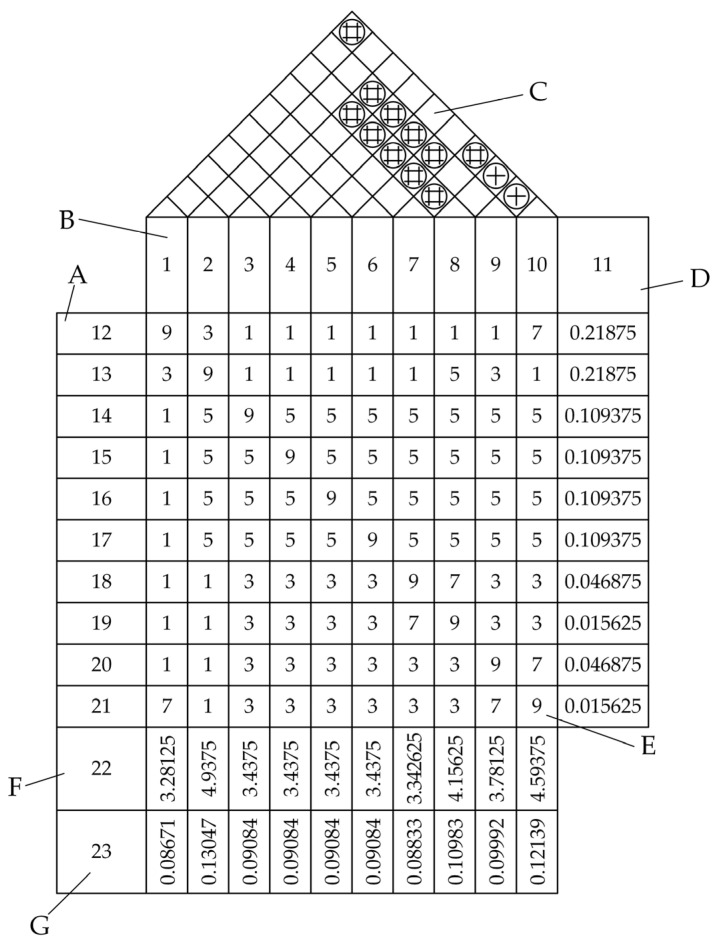
QFD quality house requirement-mapping analysis results.

**Figure 8:**
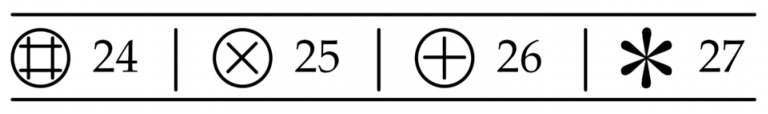
Types of pairwise correlations between technical measures.

In Figure 7, Section A outlines design requirements; Section B details technical measures; Section C presents pairwise relationships between technical measures, with corresponding meanings illustrated in Figure 8; Section D shows weight values for design requirements calculated using Formulas (2)–(9); Section E indicates relationship values between different technical measures for individual design requirements; Section F calculates technical measure importance values through Formula (10) using Sections D and E; Section G derives further analysis weights for technical measures from Formula (11). The meanings of the numbering in Figure 7 and Figure 8 are shown in Table 6.

#### 4.5.3. Comprehensive Evaluation Method for Weight Analysis Results

For the constructed QFD quality house, the demand weight analysis results can be obtained, and then the technical weight analysis results can be further analyzed. The technical weight analysis results can be combined with a fuzzy evaluation method to comprehensively evaluate the two design schemes and select the optimal design scheme.

Regarding the two design schemes generated during decoupling design, (1) the test device is arranged at the head with the interface position as the detonator section, and (2) the test device is installed at the bottom of the cartridge with the interface position as the cartridge base. Technical indicators (FR) are categorized, and in this design, all quantitative evaluation indicators are employed except for the device’s external dimensions control.

For quantitative indicators, the specified requirement value is the design range, the actual distribution function of the index is the system range *R_s_*, and the overlapping position between the design range and the system range is the common area *R_c_* [10]. The calculation formula of information *I* is

(12) $$I=\ln\frac{R_{s}}{R_{c}}.$$

Qualitative indicators need to be artificially divided into certain levels and then transformed into quantitative indicators for evaluation and analysis. This process requires the introduction of fuzzy mathematical theory; the area enclosed by the affiliation function curve of the system range is defined as the fuzzy system range *R_fs_*; the intersection of the fuzzy design range and the fuzzy system range is defined as the fuzzy public range *R_fc_* [10]. Then, the calculation method of the amount of information is as follows:

(13) $$I=ln\frac{R_{fs}}{R_{fc}}.$$

The selection of membership functions can be determined by their distribution types, which can be normal, ridge, or Cauchy distributions with nonlinear transition bands, or general linear distributions like triangular or trapezoidal shapes with linear transition bands. Based on these principles, the evaluation metrics and information values for both Scheme I and Scheme II are calculated as shown in Table 7. The amount of information $I_{w}$ of the weight analysis scheme is calculated as follows [10]:

(14) $$I_{w}=B_{j}\times I.$$

As shown in Table 7, the amount of information of Scheme II after weight analysis is 0.665, representing a 33.57% reduction compared to the 1.001 amount of information of Scheme I. According to the Information Axiom and the previous analysis, it can be seen that under the premise that both meet the test requirements, Scheme II is the optimal design scheme. The engineering design of the high-overload test device can be further realized based on the design process and functional mapping structure of Scheme II.

#### 4.5.4. Application Validation Study

**Figure 9:**
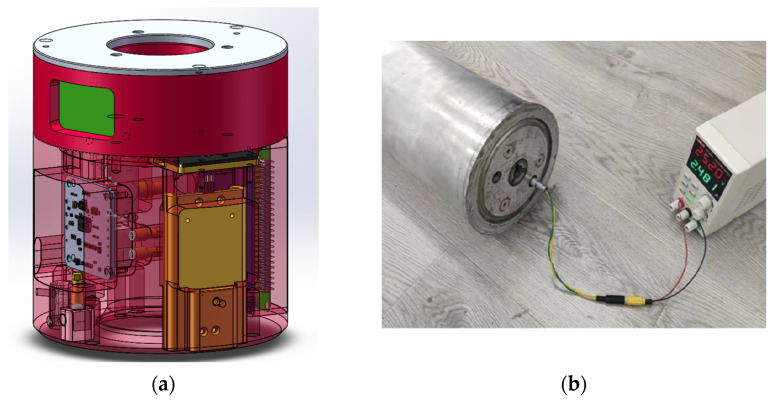
High-overload test device. (**a**) The three-dimensional modeling perspective; (**b**) The test device is charging.

We rigorously validated the proposed AD-TRIZ framework through prototype testing of Scheme II. The three-dimensional modeling perspective of the high-load test apparatus shown in Figure 9a is specifically designed for integration into high-speed drones operating under extreme dynamic conditions. The modular test core mounted on the drone’s bottom structure integrates multiple MEMS-based sensors, including the YND-YD-6050 attitude sensor, YND-YD-236-5 motion parameter sensor, YND-YD-236 vibration sensor, and the YND-YD-209 shock sensor. Data storage utilizes a high-speed solid-state drive unit, while power is supplied by a series-configured 18,650 lithium battery pack. Figure 9b depicts the charging status of the test apparatus.

**Figure 10:**
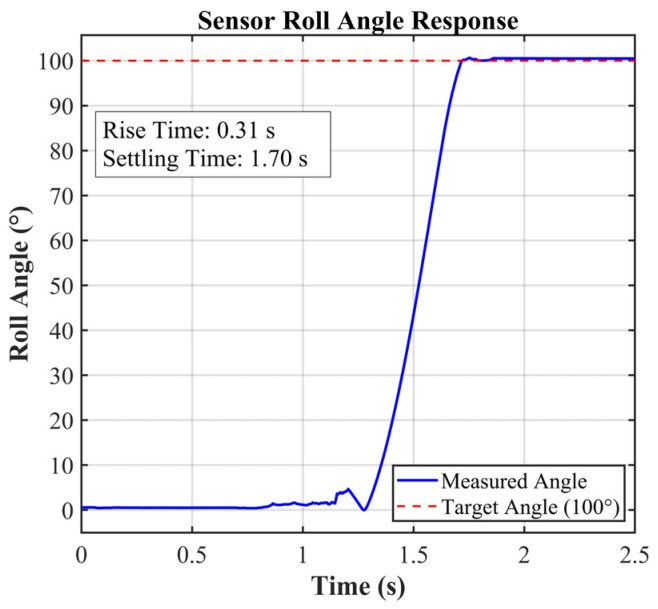
Sensor roll angle response.

Validation testing involved applying a controlled 100° roll-angle rotation using a calibrated rotational platform. The YND-YD-6050 attitude sensor recorded roll-angle changes at 1 kHz sampling frequency, with raw data stored in the onboard SSD. As shown in Figure 10, the sensor response demonstrated linear angular progression during rotation phase followed by stable 100° positioning, exhibiting minimal overshoot (±0.5°).

These experimental results confirm the AD-TRIZ-based design achieved robust operation under actual high-overload conditions. The optimized sensor integration derived from TRIZ decoupling principles eliminated structural interference issues, while the axiomatic decomposition ensured measurement fidelity during rapid transient events. The complete validation workflow demonstrates the methodology’s effectiveness in developing mission-critical test equipment for extreme environments.

## 5. Discussion

In this paper, we propose a design methodology for high-overload testing equipment based on axiomatic design and problem-solving theory. This methodology assists novice designers in completing the design of testing equipment according to standardized procedures. This method employs axiomatic design to progressively decompose design requirements within the specifications, simplifying design complexity. It addresses conflicts arising during the design process using problem-solving theory, as such conflicts often stem from functional structural conflicts. Problem-solving theory, specifically developed to address such conflicts, effectively facilitates the research and resolution of these issues. This paper leverages the advantages of axiomatic design and problem-solving theory. Additionally, by addressing the issue of inaccurate or subjective evaluations of multiple design solutions in traditional problem-solving theory, we propose a multi-level evaluation method combining subjective and objective criteria to comprehensively assess potential solutions and determine their relative merits. In terms of method validation and application examples, this paper uses the design process of a high-overload testing device as an application example. This example can guide the design process of similar testing devices and provide designers with a standardized template, enabling them to quickly develop corresponding design solutions tailored to different application directions.

## 6. Conclusions

In addressing the challenges posed by high-overload testing equipment, which traditionally relies heavily on the experience-based characteristics of designers, and the varying design methods and work efficiencies of designers with different levels of experience when tackling specific issues, in this paper, we propose a design process and evaluation method adaptable to designers of any experience level. Under this approach, researchers can focus their limited time and resources on resolving specific testing issues without needing to concern themselves with the correctness of the design process. This paper primarily combines axiomatic design (AD) with TRIZ problem-solving theory. This method employs a three-layer technical decomposition framework: requirement–parameter mapping, where overall technical specifications are gradually decomposed into independent functional requirements (FRs) and technical parameters (TPs), and a design matrix is constructed to facilitate requirement transmission; conflict identification and resolution, where based on the design matrix, conflict structures are identified through coupling analysis, and TRIZ contradiction matrices are utilized to invoke innovation principles (such as the segmentation principle and pre-compensation principle) to reconfigure design solutions; and procedural quantitative evaluation, where a multi-criteria evaluation system covering reliability, accuracy, cost, and manufacturability is established and the entropy weight–AHP method is combined to quantify the comprehensive score of the procedure. A design example demonstrates the specific design process of this method, and an overload testing device was developed using this design method. In actual use, the device can effectively complete testing tasks and significantly shorten the process of modifying preliminary designs. This method effectively addresses issues caused by excessive reliance on empirical methods in traditional design: By replacing trial-and-error iteration with AD’s “zigzag decomposition path”, the development cycle is significantly shortened while improving conflict resolution efficiency. The TRIZ toolkit reduces the time required to resolve typical technical conflicts, strengthens the objective evaluation of design solutions, and minimizes design preference biases through integrated evaluation models. The resulting modular high-load testing equipment framework validates the engineering applicability of this method. This framework provides a quantifiable, reusable system paradigm for the design of extreme environment testing equipment, and its “demand-driven conflict resolution–comprehensive evaluation” logic can be extended to the development processes of similar equipment.

## Figures and Tables

**Table 1:** Advantages and limitations of different intelligent design methods.

Method	Core Indicators	Merit	Limit
Axiomatic Design	Ensures functional independence through design matrices. Information axioms require minimizing design uncertainty and improving system controllability [21].	Provides a structured problem decomposition framework to support complex system design. Uses “zigzag mapping” to clarify the relationship between function and structure, reducing iteration costs [22,23].	Difficult to handle highly coupled matrices. Reliance on designers’ experience to resolve conflicts, lack of innovative tools, insufficient adaptability to dynamic user demands.
Theory of Inventive Problem Solving	Forty invention principles to resolve technical conflicts [24]. Conflict matrix conversion engineering parameters. Idealized final results.	Efficiently resolves technical conflicts. Provides a systematic innovation model to shorten the R&D cycle. Supports cross-domain applications [25,26,27].	Weak multi-conflict processing capabilities. Complex abstract parameter conversion. Reliance on patent libraries limits applicability in emerging fields.
Robust Design	Noise interference resistance. Quality loss function [28].	Reduces warranty costs and scrap rates. Improves product performance consistency and reduces sensitivity to noise factors.	High design complexity. Difficult to resolve structural conflicts.
Design For Six Sigma	Six Sigma quality level. Customer satisfaction.	Systematically integrates demand management to reduce later modifications. Improves product reliability and manufacturability. Reduces development cycle and costs through structured processes [29].	Implementation depends on cross-functional team collaboration. Strict data integrity requirements.

**Table 2:** Basic requirements of test functions.

Number	Primary Performance Indicator	Secondary Performance Indicator
1	Physical properties (CA1)	Device size requirements (CA11)
		Device quality control (CA12)
2	Test characteristics (CA2)	Motion characteristics measurement (CA21)
		Posture characteristic measurement (CA22)
		Vibration characteristics (CA23)
		Impact characteristics measurement (CA24)
3	Power supply characteristics (CA3)	Power module life (CA31)
		Power module capacity (CA32)
4	Data transport and storage performance (CA4)	Data storage capacity (CA41)
		Data read capability (CA42)

**Table 3:** Conflict matrix.

	Improvement Parameters	No. 2	No. 9
Deterioration Parameters			
No.32		—	35, 13, 8, 1
No.36		35, 8, 26, 39	—

**Table 4:** The underlying inventive principles.

Number	Principle of Invention	Principle and Innovation Direction
1	Segmentation	To divide something into separate or detachable parts
2	Taking Out	To extract the “interference” part or characteristics from the system, or only extract the required part or characteristics
8	Merging	The merging of objects or operations that are similar or adjacent in space or time
13	The Other Way Around	To perform the opposite of the original action, to switch the static and dynamic state or up and down relationship of the original parts
26	Copying	To replace expensive, fragile objects with simple, inexpensive replicas
35	Parameter Changes	To change the physical properties of an object, to change the concentration or viscosity of an object, to change the flexibility of an object or to change the temperature of an object
39	Inert Atmosphere	To replace the normal environment with an inert gas environment, add inert or neutral additives to the object or use vacuum packaging

**Table 5:** The relative importance scoring criteria.

Number	Relative Importance	Meaning
1	1	The importance is the same
2	3	The former is slightly more important
3	5	The former is important
4	7	The former is significantly more important
5	2, 4, 6	The importance lies between the two adjacent levels above
6	1, 1/2, 1/4, 1/6	The two indicators are compared to each other in the opposite order

**Table 6:** Numbering comparison.

Numbering	Evaluation Indicators	Numbering	Evaluation Indicators
1	Control device external dimensions	15	Attitude characterization measurements
2	Device weight adjustment	16	Vibration characterization measurement
3	Motion test performance	17	Impact characteristics measurement
4	Attitude test performance	18	Power module lifetime
5	Vibration test performance	19	Power module capacity
6	Impact test performance	20	Data storage capacity
7	Power module lifetime	21	Data reading capability
8	Power module capacity	22	Technical importance Aj
9	Data storage capacity	23	Technology weighting Bj
10	Data interface transmission protocol	24	Negative impact
11	Weighting of design requirements W	25	Particularly negative impacts
12	Device size requirements	26	Positive impact
13	Device quality control	27	Particularly Positive impact
14	Motion characteristics measurement		

**Table 7:** Evaluation indicators, factor set, and information volume of test device design scheme.

Set of Evaluation Indicators	Scope of Design	Meaning	Scheme I	Scheme I Amount of Information	Weight Analysis Scheme I Amount of Information $I_{w}$	Scheme II	Scheme II Amount of Information	Weight Analysis Scheme II Amount of Information $I_{w}$
Control of device dimensions	Good	The importance is the same	Excellent	0.357	0.031	Excellent	0.511	0.044
Adjusting the device counterweight	0.95	The former is slightly more important	0.96	1.609	0.210	0.98	0.511	0.067
Exercise test performance	0.95	The former is important	0.98	0.511	0.046	0.98	0.511	0.046
Posture test performance	0.95	The former is significantly more important	0.97	0.916	0.083	0.98	0.511	0.046
Vibration test performance	0.95	The importance lies between the two adjacent levels above	0.98	0.511	0.046	0.98	0.511	0.046
Impact test performance	0.95	The two indicators are compared to each other in the opposite order	0.96	1.609	0.146	0.96	1.609	0.146
Power module life	0.9		0.95	0.693	0.061	0.95	0.693	0.061
Power module capacity	0.9		0.93	1.204	0.132	0.94	0.916	0.101
Data storage capacity	0.9		0.92	1.609	0.161	0.98	0.223	0.022
Data interface transmission protocol	0.9		0.95	0.693	0.084	0.95	0.693	0.084
Total				9.712	1.001		6.689	0.665

## Data Availability

The original contributions presented in the study are included in the article; further inquiries can be directed to the corresponding author.

## Abbreviations

The following abbreviations are used in this manuscript:

AD	Axiomatic Design
TRIZ	Theory of Inventive Problem Solving
AHP	Analytic Hierarchy Process
QFD	Quality Function Deployment
DP	Design Parameter
FR	Functional Requirement
PV	Process Variable
CI	Consistency Index
CR	Consistency Ratio
MEMS	Micro-Electro-Mechanical Systems
RI	Random Index
SSD	Solid State Drive
UAV	Unmanned Aerial Vehicle
USB	Universal Serial Bus
*Rs*	The actual distribution function of the index is the system range
*Rc*	The overlapping position between the design range and the system range is the common area
*Rfs*	The area enclosed by the affiliation function curve of the system range is defined as the fuzzy system range
*Rfc*	The intersection of the fuzzy design range and the fuzzy system range is defined as the fuzzy public range

## Appendix A. The 39 Engineering Parameters of TRIZ

**Table A1:** The 39 engineering parameters of TRIZ.

No.	Title	No.	Title	No.	Title
1	Weight of moving object	14	Strength	27	Reliability
2	Weight of stationary object	15	Duration of action by a moving object	28	Measurement accuracy
3	Length of moving object	16	Duration of action by a stationary object	29	Manufacturing precision
4	Length of stationary object	17	Temperature	30	External harm affects the object
5	Area of moving object	18	Illumination intensity	31	Object-generated harmful factors
6	Area of stationary object	19	Use of energy by moving object	32	Ease of manufacture
7	Volume of moving object	20	Use of energy by stationary object	33	Ease of operation
8	Volume of stationary object	21	Power	34	Ease of repair
9	Speed	22	Loss of energy	35	Adaptability or versatility
10	Force	23	Loss of substance	36	Device complexity
11	Stress or pressure	24	Loss of information	37	Difficulty of detecting and measuring
12	Shape	25	Loss of time	38	Extent of automation
13	Stability of the object’s composition	26	Quantity of substance/the matter	39	Productivity

## Appendix B. Example of Amount of Information Calculation

Example (Adjusting the device counter-weight for scheme I): The design range requires the error between tested and standard values to be within 5%. The system range is (0.95, 1), and for Scheme I, the tested value is 0.96 (error 4%), so the common range is (0.95, 0.96). Thus, $I=\ln\left(1-0.95\right)/\left(0.96-0.95\right)=1.609$.

Example (Control of device dimensions for scheme I):Indicators “device dimension control” involve randomness and subjectivity, evaluated using fuzzy mathematics. Based on a five-level evaluation set {Poor, Fair, Medium, Good, Excellent} scaled as {1, 2, 3, 4, 5}, the average scoreV is computed from expert ratings.

Ten experts rated it: 6 as Excellent (5), 2 as Good (4), 2 as Medium (3). Thus, $V=\left(6\times 5+2\times 4+2\times 3\right)/10=4.4$.

For benefit-type indicators (higher values better), $R_{fs}=5-3=2$ (fuzzy system range) and $R_{fc}=V-3=1.4$ (fuzzy common range for V = 4.4). Hence, I=ln(2/1.4)=0.357.

Therefore, the weight analysis amount of information $I_{w}$ is $I_{w}=B_{1}\times I=0.08671\times 0.357=0.0310$.
